# Involving youth with intellectual and/or developmental disabilities as collaborators in a comparative effectiveness trial: A community-engaged research approach

**DOI:** 10.1016/j.conctc.2024.101395

**Published:** 2024-11-22

**Authors:** K.L. Berg, D Herrman, L Bernard, C.S Shiu, I Mihaila, C Arnold, K Acharya, T.R.G Gladstone, C Danguilan, H Gussin, P Perez, A Herrman, S Aaron, A Thornton, M Gerges, C Patriarca, J.J Pak, B.W Van Voorhees

**Affiliations:** a Department of Disability and Human Development, College of Applied Health Sciences, University of Illinois at Chicago, Chicago, IL, 60612, USA; b Department of Pediatrics, College of Medicine, University of Illinois at Chicago, Chicago, IL, 60612, USA; c Doctor of Physical Therapy Program, College of Health and Human Sciences, Northern Illinois University, DeKalb, IL, 60115, USA; d University of California Los Angeles, Los Angeles, CA, 90095, USA; e Brown University, Providence, RI, 02912, USA; f The University of Illinois Division of Specialized Care for Children, Chicago, IL, 60607, USA; g The Arc of Illinois, Mokena, IL, 60448, USA

## Abstract

**Background:**

Practices to include youth with intellectual and/or developmental disabilities (IDD) are necessary to design and implement research that specifically meets the behavioral health needs of this population. This article describes a protocol for engaging youth with IDD as collaborators in a comparative effectiveness clinical trial using a community-engaged research (CEnR) approach.

**Methods:**

Our engagement protocol, guided by the Community Engaged Research (CEnR) Framework, emphasized harm avoidance, accessibility, demonstrated value, capacity bridging and co-learning, shared power and equity in decision-making, accountability and respect, and transparent communication. We involved seven youth with IDD in a Youth Advisory Committee (YAC) and four youth with IDD in a Summer Scholars program, ensuring consistent and structured engagement throughout the study.

**Results:**

Youth with IDD maintained high levels of engagement in both the YAC and Summer Scholars Program with 100 % retention across two years. Youth used multiple modalities to provide feedback on aspects of the research project, resulting in study modifications, the co-development of products, and tangible improvements in the accessibility and relevance of the study for youth with IDD.

**Conclusion:**

Researchers and clinicians seeking to engage the historically underserved population of disabled youth in clinical trial research can leverage our findings to enhance the accessibility and inclusivity of their studies.

## 1 Background

Youth with IDD experience high rates of anxiety and depression [1,2] and are significantly less likely than their non-disabled peers to receive services to address their behavioral health needs [3]. Although estimates vary based on age and diagnosis, approximately 1 out of 4 youth with disabilities have an unmet behavioral health care need [4]. Untreated depression and anxiety in youth with IDD are associated with long-term negative impacts on health, functioning, independence, and transition to adulthood outcomes [1]. [2,3,5].

A major barrier to accessing behavioral health services for youth with IDD is the limited availability of evidence-based, community-informed interventions [6]. An estimated 75 % of clinical trials have explicitly or implicitly excluded individuals with IDD, neglecting their priorities in the research process and hindering the development of tailored interventions [7,8]. This exclusion stems from researchers' lack of knowledge and resources, communication barriers [9], ethical concerns regarding informed consent [10], perceptions that accommodations are time and cost-intensive [11], and prevailing ableism and stigma [12]. Consequently, many treatments have been developed without considering the unique needs of youth with IDD, resulting in a gap in evidence-based practices and perpetuating disparities in healthcare and social services [13].

In response to these historic inequities, US federal agencies and health research organizations are prioritizing inclusive research with people with IDD. The National Institutes of Health recently designated people with disabilities as a health disparities population, revised its mission to prioritize their inclusion in clinical research, and initiated new programs and funding to advance knowledge in this area [14]. Additionally, the Patient Centered Outcome Research Institute (PCORI), the foremost funder of patient centered clinical research in the US, has advanced an agenda that prioritizes research involving people with IDD, and disseminated guides for engaging people with IDD as community collaborators [15]. Inclusive research treating people with IDD as collaborative partners [16] leads to more relevant, impactful clinical outcomes and a heightened sense of ownership in the research process [17,18]. Studies that have engaged youth with IDD using inclusive research practices [12,18,19] have generated positive outcomes, including meaningful community participation, transferable knowledge, leadership, skills, and a sense of empowerment.^17.9^ However, most of these studies were not focused on behavioral healthcare delivery or intervention approaches [13]. Additional studies involving youth with IDD are needed to evaluate the feasibility and impacts of inclusive research in large-scale clinical trials of behavioral health interventions and care delivery for this population.

This article aims to describe (1) the protocol for engaging youth with IDD as collaborators in the BEhavioral Health Stratified Treatment (BEST) study, a PCORI-funded comparative effectiveness trial [20], including methods to recruit, orient and engage youth as co-leaders in the study, and; 2) initial findings from engagement activities during the early phases of the BEST study. Researchers and clinicians can leverage our findings to enhance the accessibility and inclusivity of their clinical studies. \

## 2 Methods

### 2.1 Overview of the BEST study

The BEST Study is a randomized clinical trial evaluating the effectiveness of an integrated behavioral health care coordination model compared to standard care coordination for improving depression, anxiety, quality of life, and healthcare transition outcomes in youth with IDD, ages 13–20 [21]. Core elements of the BEST model include a treatment algorithm for categorizing depression and anxiety risk, stratified mental health interventions, and a system for communication and data exchange between care coordinators and BEST personnel (Berg et al., 2024) [21]. The multidisciplinary BEST team, selected for their expertise in Social Work, Disability Studies, Rehabilitation Sciences, Developmental & Behavioral Medicine, Psychology, and Methodology, also brings strong community ties, including relationships with disability advocacy groups. A crucial member of the team, the director of community engagement, leads efforts to recruit youth with IDD, organizes advisory meetings, and co-leads the Summer Scholars program with the project director.

From its inception, the BEST Study has employed a Community-Engaged Research (CEnR) approach, actively involving youth with IDD throughout the research process [22]. CEnR is defined as an inclusive participation process that promotes mutual respect of values, strategies, and actions for authentic partnership with people affiliated with or self-identified by geographic proximity, special interest, or similar situations to address specific issues [23]. The NORC CeNR framework [24], adapted from the Agency for Toxic Substances and Disease Registry Principles of Community Engagement [25] and other evidence-based models, consists of 6 principles for community engagement: (1) **avoidance of harm**, working with team members to understand the broader implications of the research and avoid marginalization of the community by recognizing biases, listening to community expertise, and preventing perpetuation of negative interactions; (2) **accessibility and demonstrated value**, showing all team members that their time and contributions are valued using flexible and equitable methods of engagement; (3) **capacity bridging and co-learning,** supporting all team members in learning from each other and engaging in bi-directional feedback; (4) **shared power and equity in decision making**, encouraging equitable cooperative decision making at all research stages; (5) **accountability and respect**, facilitating equitable methods to incorporate community expertise into decision-making, promote commitment, and address discord; and (6) **transparency and open communication**, leading to honest conversations about power dynamics, decision-making, resources, challenges, data, findings, and dissemination amongst researchers and community members [24].

**Fig. 1:**
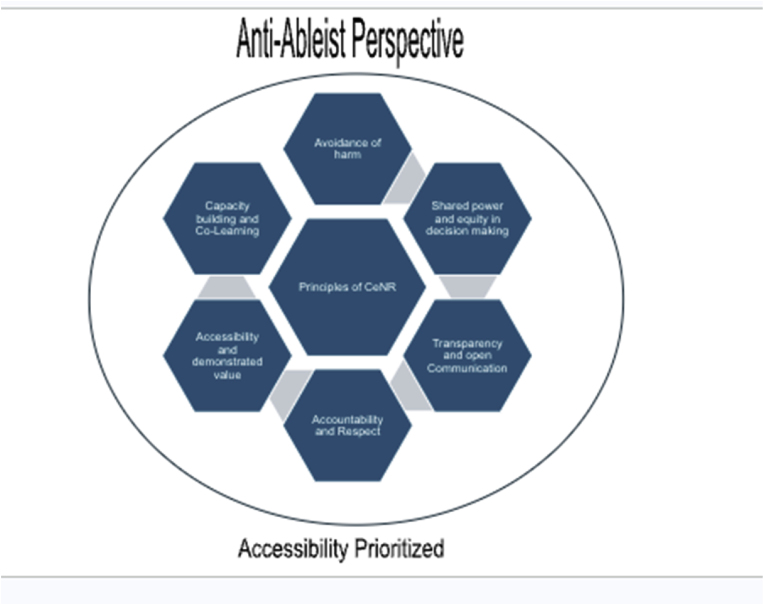
CEnR principles with engagement protocol in BEST project.

This approach aligns with PCORI's principles of stakeholder engagement [26] and empowers historically marginalized groups, such as youth with IDD, to shape interventions and influence organizational improvements that directly impact their community. Furthermore, this framework adheres to the disability community's mantra, “nothing about us without us,” [27] and responds to calls for greater engagement and community participation in research among youth with IDD. The following sections describe the engagement protocol and how CEnR principles (Fig. 1) were embedded within these processes to more meaningfully involve youth with IDD in the research (Table 1).

**Table 1:** CeNR principles and project examples.

CeNR Principles	Examples of Implementation Strategies
**Avoidance of Harm**	The research team participated in activities to increase their disability cultural competence ● Reflective activities and discussions about previous assumptions of disability ● Discussions on disability cultural humility included: ● Promotion of disability pride and success of disability community
**Accessibility & Demonstrated Value**	● Prioritized and implemented anti-ableist practices ● Consulted experts (ARC of Illinois, Great Lakes ADA Center) for accessibility and technology use ● Universal design approach (e.g., captions for Zoom, plain language, SymbolStix) ● Individualized accommodations (e.g., caregiver assistance for recording feedback, shortened interview sessions) and regular check-ins
**Capacity Bridging & Co-Learning**	● Research skills training sessions for youth ● Mentorship on conference abstract development, presentation skills
**Shared Power and Equity in Decision-Making**	● Co-created a structure for the YAC meetings and summer scholar Program. ● Orientation of YAC and summer scholars ● Co-established protocols for shared decision-making ● Recorded meetings and decisions to share and member check with youth
**Accountability & Respect**	● Co-developed guidelines to ensure safe, respectful, and inclusive participation. ● Consensus-building techniques to guide decision-making processes
**Transparency & Open Communication**	● Regularly scheduled meetings to discuss study progress and gather feedback from youth ● Protocols for recording and transcribing meetings with chat transcripts to document youth feedback and its influence on project decision-making ● Accuracy and member checking by sharing meeting notes and auto-generated transcripts ● Regular reporting of study updates and project adaptations ● Multiple means of communication: email summaries, status updates at meetings, and social media/website updates ● Regular evaluations and check-ins to address any challenges

### 2.2 Adoption of an anti-ableist perspective to avoid harm

At the core of the BEST intervention is an anti-ableist approach to engaging youth with IDD. Ableism, or systemic oppression based on societal standards of normalcy, ability, and health [28], has historically led to the stigmatization and unequal treatment of people with IDD, including their exploitation in prior research studies [29]. In recognition of this history, the research team engaged in reflective activities and discussions to enhance their “disability cultural competence” in areas such as biomedicine, history, accessible technology/design, legislation, and social justice [30]. Accessibility was also recognized as a core component of the project's anti-ableist, inclusive research approach [[31], [32], [33]]. Before engaging youth, the team consulted experts from the ARC of Illinois and the Great Lakes ADA Center to evaluate the accessibility of study materials, technology platforms, and tools, and to create multiple feedback mechanisms (e.g., captions for Zoom, plain language, SymbolStix). Procedures were also established for one-on-one meetings and regular check-ins with youth to identify needs and monitor the implementation of individualized accommodations (e.g., caregiver assistance for recording feedback shortened interview sessions). The team also set up procedures to compensate youth collaborators directly, recognizing their value, agency, and expertise as equal contributors.

### 2.3 Establishing governance structures and practices to promote shared decision making, open communication and accountability

The research team established practices for shared governance based upon best practices identified in the literature and informed by disabled community advocates who partnered in developing the BEST study proposal [32]. The process of creating and implementing shared governance involved 1) creating the advisory committees and Summer Scholars program; 2) member orientation; 3) establishing protocols for shared decision-making; 4) creating a structure for the YAC meetings and Summer Scholars; and 5) establishing protocols for transparency and open communication.

#### 2.3.1 Creating advisory committees and the Summer Scholar program

Initially, we established two community advisory committees, a youth and families (YFA) committee and a general advisory committee (CAC). The YFA was designed to solicit youth and family feedback on all aspects of the study, including proposed language, instrument development, recruitment and retention strategies, intervention adaptation, implementation, and dissemination. In addition to the YFA and CAC, youth with IDD were recruited to consult on the project as Summer Scholars during the pre-implementation and early implementation phases. Summer Scholars completed a paid internship with the University of Illinois-Chicago Department of Pediatrics, in which they piloted and provided additional feedback on specific aspects of the project and co-created multimedia tools to enhance the accessibility and usability of intervention and assessment components (see section 2.3.5). *Recruiting Community Members.* In alignment with CEnR principles [24], our objective was to engage diverse advisory members that reflected the lived experiences of the community served by the project. During the first phase of the project, members of the research team reached out to disability rights organizations, community partners, parents, and youth to recruit committee members representing diverse backgrounds. English and Spanish language flyers and promotional materials were developed and adapted by team members with disabilities in collaboration with marketing experts and the ARC of IL, to maximize accessibility and appeal to our target population. Staff members with established relationships with local and regional organizations (i.e., Institute on Disability and Human Development, the Division of Specialized Care for Children, Access Living of Metropolitan Chicago, Disabled People of Color Coalition, ARC of IL) shared promotional materials for dissemination within the organizations’ networks. The Director of Community Engagement contacted potential recruits to explain the project and the role of the YFA, gauge interest, and identify support needs for participation. Information about compensation ($25.00 per meeting) and platform (web-based; Zoom with access to an interpreter, transcripts, and visual aids) was also shared to incentivize participation. Upon recruitment, youth were encouraged to share digital flyers with partners and on social media to attract other youth with IDD to join and participate in study-related opportunities, including the Summer Scholar program. Seven YAC members and four Summer Scholars were ultimately recruited for the project. *Youth-Led Changes to the Advisory Committees.* During the first YFA meeting, youth requested that the team prioritize their voices by establishing a separate advisory committee exclusively for youth. In response, the team established the Youth Advisory Committee (YAC) and encouraged family members to join the Community Advisory Committee (CAC) instead. To support collaboration and communication across the advisory groups, two youth served as members of both the CAC and the YAC. All meetings were held virtually through Zoom.

#### 2.3.2 Orientation to YAC and Summer Scholars Program: promoting capacity bridging and accountability

Orientation served as an important stage in developing a shared vision for the research partnership and a culture of mutual respect. Before the initial YAC and Summer Scholars meeting, individual meetings were conducted to discuss roles and responsibilities of youth members and staff. Specific to the YAC: During the first meeting, the group co-developed guidelines to ensure safe, respectful, and inclusive participation. Youth then led development of a pictorial logo representing the team and project's shared values and vision (See Appendix A). For the Summer Scholars program orientation, the project director and Summer Scholars met one-on-one to collaboratively establish internship guidelines based upon individual needs and preferences of Summer Scholars, and the needs and capacities of the project. Orientation also involved a primer on the BEST study and research skills training sessions **(Capacity Bridging & Co-Learning)** to empower youth with skills and understanding to participate in decision-making.

#### 2.3.3 Establishing protocols to promote equity in decision-making

During orientation and throughout the project, we created and adapted procedures to ensure equitable participation and address power differentials that could impede shared governance. For example, we implemented a “fishbowl” process during CAC meetings in which youth members were first shared their thoughts and ideas while other CAC members listened before responding. Additionally, we scheduled CAC meetings one week before the YAC meetings, allowing CAC member recommendations to be evaluated by the youth. Finally, we instituted consensus-building techniques to guide decision-making processes during the YAC and small group Summer Scholar meetings to ensure equitable collaboration and productive resolution of differences (**Accountability and Respec**t).

#### 2.3.4 Creating a structure for the YAC meetings and Summer Scholars Program

Structure of the YAC meetings. First, all YAC members received electronic agendas in advance of quarterly meetings to enable preparation and access to comprehension supports (i.e., team-facilitated supports, including calls or one-on-one meetings). Agendas were written in plain language, supplemented with visual imagery (i.e., Symbol Styx), and edited by the ARC of IL to enhance accessibility and comprehension (see Appendix A). Second, YAC meetings opened with review of group guidelines, followed by a brief check-in activity whereby the youth introduced themselves, provided updates, and shared their favorite seasonal activity. This routine fostered a welcoming environment, encouraged group rapport, and provided an opportunity to address any engagement challenges youth might be experiencing. Third, project updates and study components were presented to youth for review and input. These presentations/updates were developed by the team and reviewed and adapted by the ARC of IL to maximize universal accessibility. Fourth, youth were encouraged to provide feedback on the presentation using multiple mechanisms, although oral and written feedback using the Zoom chat function were most common. Some youth later provided written or oral feedback, after taking time to process and evaluate the information. Skilled staff helped facilitate discussions, ensuring all voices were heard. Brainstorming was employed to generate free flow of ideas from diverse participants, while informal voting procedures included verbal input, anonymous polls, and the Zoom chat function. Lastly, meetings concluded with a summary of key points and decisions, an open floor for final questions and comments, an outline of next steps, confirmation of details for subsequent meetings, and an expression of appreciation. The overall structure of the YAC meetings reflected the CEnR principles by ensuring mutual respect, accountability and equitable decision-making through its accessible agendas, inclusive feedback mechanisms, and facilitated discussions that valued all voices.

*Structure of the Summer Scholars Program*. The Summer Scholars program was primarily active during the early implementation phase of the study, with most participants engaged for 3–4 months. The program provided a paid, structured opportunity for youth with IDD to contribute to developing and adapting BEST study materials, offering a valuable experience that could enhance their career development and strengthen their resumes. The project director and the director of community engagement worked closely with the scholars throughout the program. Scholars typically met with study staff weekly or biweekly via Zoom to pilot and provide feedback on specific study components. Study components were typically reviewed and tested across multiple micro-sessions, either in one-on-one meetings with a staff member or in small groups. Plain language, visual aids, repetition, and coaching were employed to improve accessibility and comprehension of study components. Similar to the YAC, Summer Scholars provided feedback in through oral, written, and caregiver-assisted communication. Summer Scholars also engaged in product development according to areas of interest, including the co-creation of comics, animation, videos, and clinical scenarios. The internship structure reflects the CEnR principles in several ways: 1) ensuring accessibility and demonstrated value through individualized support and plain language; 2) facilitating capacity bridging and co-learning with coaching/skills development in one-on-one sessions; 3) promoting shared power and equity in decision-making by aligning tasks with interests and integrating youth feedback on study components; 4) maintaining accountability and respect through paid engagement and project updates; and 5) fostering transparency and open communication via regular, structured meetings.

#### 2.3.5 Establishing protocols for transparency & open communication

The YAC and Summer Scholars program facilitated transparency and open communication by providing a platform for regularly scheduled meetings to discuss study progress and gather feedback. Additional protocols for recording and transcribing meetings were instituted to document feedback and its influence on project decision-making. All meetings with youth were video and audio recorded with auto-generated transcription. Chat transcripts and meeting notes were included as documentation of youth participation. Team members reviewed auto-generated documents and performed member checking to ensure accuracy. Procedures to regularly report meeting minutes, study updates, and log project adaptations to our YAC members were also implemented, including email summaries, status updates at meetings, and social media/website updates. Finally, regular check-ins with YAC members and Summer Scholars were conducted to evaluate the shared decision-making process and address any challenges. These steps demonstrated the project's commitment to honesty and accountability, prerequisites to fostering trust and engagement between youth with IDD, a historically marginalized population, and researchers.

## 3 Results

### 3.1 Engagement of youth

Team members’ involvement with disability organizations and record of anti-ablest work (i.e., “avoidance of harm”) were key factors in successfully recruiting and engaging youth with IDD as collaborators. Team members embedded and allied with disability community organizations facilitated the rapid recruitment of diverse youth with IDD by leveraging established trust and networks within these communities, ensuring inclusive and effective outreach. Word of mouth was the most effective means to recruit youth for the YAC and the Summer Scholars program. The YAC (N = 7) began meeting quarterly in May 2022, meeting nine times, and, as of July 2024, has retained 100 % of its members. Similarly, the Summer Scholars program recruited four youth with IDD, all of whom completed the internship and produced creative materials. While we intentionally recruited collaborators from diverse community groups to enrich the diversity of our YAC and Summer Scholars group, sociodemographic data about the youth was not collected, as they were collaborators and contributors to the broader research rather than study participants.

The principle of accessibility and demonstrated respect was crucial for maintaining participation and engagement in the YAC and Summer Scholars program. Virtual meetings were offered evenings and weekends to accommodate youth schedules. Also, monetary compensation was tied to participation in meetings for YAC and hourly work for Summer Scholars. This strategy communicated respect for the youths’ time and expertise, possibly assisting in sustaining engagement. Attendance rates were high among youth (85–100 %). Also, study feedback mechanisms were adapted and flexible to meet the needs of youth (i.e., oral, written, and assisted communication options during meetings, procedures to enlist caregiver support, one-on-one Zoom sessions), which enhanced engagement. For example, one youth with significant communication needs requested that her caregiver be allowed to relay her feedback after she had time to review and process the information. By offering this youth greater flexibility, she could relay important feedback (about what she liked, the process of using the program, her overall experience with the intervention) on intervention content.

### 3.2 Learning from youth feedback

The diverse feedback from youth with IDD on multiple aspects of the project exemplified shared power and equity in decision-making by ensuring their perspectives influenced and improved the project's development and outcomes. As of July 2024, there were over 47 meetings and 55 feedback events involving youth with IDD.

The following section highlights types of youth feedback and its impact on the project, as documented through the team's records and audit trails (see Section 2.3.5).

#### 3.2.1 Youth improved the accessibility of study materials

The involvement of youth with IDD in the YAC resulted in tangible improvements to the accessibility of BEST study materials. A key concern raised was the comprehension or reading level of study materials (Table 2). Youth feedback led to enhancements such as incorporating SymbolStix and other visual aids to communicate complex concepts related to cognitive behavioral therapy strategies and other intervention components. In one instance, the youth asked researchers to change a thumbs-down emoji to a “shrugging picture” to better convey the concept of “not knowing.” Similarly, a SymbolStix image intended to represent "tired/yawning" was misinterpreted as "singing into a microphone," leading to the replacement of the image with one that better communicated "feeling tired." Finally, youth reported that study participants might perceive the tone of certain intervention materials differently than intended. In this case, all capital letters were used in a slide to improve visual accessibility. The youth disagreed, suggesting that all caps would be viewed as “yelling,” ultimately prompting changes to the font that improved visibility without using capital letters.

**Table 2:** Examples of youth or youth-assisted feedback.

Feedback on Appeal and Relevance of Intervention Materials	*[Caregiver] She liked the slide about disability pride. Even took a picture of it. She liked choosing the options on the “feelings” slides. It also prompted her to ask what some things meant, like walking on air. She also liked filling in the blanks on some of the pages. She said it was fun.*
Feedback on the Accessibility of Study Materials/Interventions	*Youth: Number 2. Don't do all caps. I wouldn't. Well, okay, like, because in (it's) the old school, and that we're taught in our school, all caps are shouting (at) people*.
Feedback on Accessibility of Study Materials/Intervention	*Youth: It looks like they're singing on microphone* *Facilitator: That's interesting! So maybe a different picture that shows tired … But yeah … I could see it also could be like, you're holding a microphone up and like they're singing … Instead of yawning, it could be confusing … If there's just the lower hand on like this, does that look more like a yawn, or does it still look like singing to you? You like it better without that hand up?* *Youth: Yeah.*
Feedback on Accessibility Of Study Materials/Intervention	*Youth: Maybe have like different variations of an explanation set out like one that's has like more complex explanations versus one that has basic explanations and of course, you can be in between, but like maybe you can do it based on like reading labels like third-grade reading level, etc.*
Feedback on Appeal and Relevance of Intervention Materials	*Some of the some of the youth ones [workshops] I feel like could be opened up to parents … I want to get more clarity to answer like the sexual health one. I think would have a similar workshop but from the lens of like your disabled children are allowed to be romantically … Here's how you support Xyz and regarding the like safe sexual health practices. I don't think it has to be so stark of like youth versus parents.*
Feedback on Consent and Assessment	*Youth: I (would) want that [mandatory reporting requirement] expressed very vividly in the survey. You know what I mean? Because, like what you don't want to have happen is for a teen not to know. [They] tell you, and then have them been blindsided.* *Facilitator: That's something we also talk about at the consent meeting. The summary that we might have to tell other people things that we learn. But I think you you're right, … we need to have that reminder at the start. So they know, it's top of mind that we might have to share things we learn from them in their interview.*
Feedback on Appeal and Relevance of Intervention Materials	“*We've stigmatized everything around mental health … you're either crazy or you're normal ….we wanna make sure that we tell the [teen] like, no there's nothing wrong with you like you are not broken like it's not you didn't do anything wrong.”*

#### 3.2.2 Youth improved the relevance of intervention materials

Youth helped co-create multimedia materials, including comic strips, artwork, and videos, to improve the appeal of study materials (See Appendix A). For example, the youth brainstormed cartoon scenarios, recorded video stories using smartphones, and utilized technology to create imagery and design characters for the cartoons and other study materials. Another way youth informed the project was by requesting and, in many cases, co-creating content for Tier 1 interventions, including workshops on sexuality and safe sex practices as well as multimedia content on disability stigma and preferred language for online modules (See Table 2).

#### 3.2.3 Youth improved the consent and assessment processes

Youth highlighted the need for greater clarity in the consent and assessment process. For example, during a YAC meeting, the facilitator explained the limits of participant confidentiality and why the research team might need to inform caregivers or authorities if participants disclose certain types of information, such as suicidality, during an assessment. This prompted a conversation about allowing youth to decide what should be disclosed to caregivers, which led to further dialogue between facilitators and youth about the legal requirements for mandatory reporting in specific situations. This exchange led to valuable insights on the need for clarity in the consent and assessment processes, yielded improvements in our consent script, and fostered greater shared understanding regarding project goals, legal or ethical requirements, and privacy concerns (See Table 2).

#### 3.2.4 Building capacity through creation of study products

As a result of their involvement in the YAC and Summer Scholars programs, the youth co-created a variety of products for the study, including the study logo, videos for online modules, comic strips for workbooks, artwork, and scripts/scenarios for online modules and group sessions (Appendix A). In addition, two members contributed to drafting and finalizing research presentations at conferences, and one youth co-presented at an international conference. One youth was invited to join a scientific panel due to her involvement with the BEST project, while another was admitted to college and plans to study film.

### 3.3 Limitations

Several limitations may have impacted our engagement protocol and youth input. First, although the team purposely recruited youth from a diverse network of organizations, the YAC and Summer Scholars may not fully represent the broader population. Second, study materials were only available in English and Spanish. Third, feedback from youth was typically not anonymous and may have been influenced by social desirability. To mitigate this risk, staff used evidence-based strategies [[33], [34], [35]] to foster trust among youth, including peer facilitation, rapport building, tech-based options to provide feedback, guidelines for constructive discussions, and resolution of disagreements. Finally, youth collaborators were not involved in the proposal writing and could not influence certain aspects of the study due to IRB (i.e., mandatory reporting for youth suicidality) and funder requirements.

## 4 Conclusion

This article presents a protocol for involving youth with IDD in a clinical trial testing the comparative efficacy of an integrated behavioral health care coordination model vs. a standard care coordination model. By applying the core principles of CEnR – avoiding harm through anti-ableist practices, promoting accessibility and demonstrating value, bridging capacity and co-learning, sharing power and equity in decision-making, being accountable and respectful, and having open, transparent communication – the protocol facilitated an iterative collaboration between youth and researchers. This approach strengthened connections, deepened mutual understanding, and fostered shared leadership, enhancing the project's procedures, outcomes, and alignment with community needs and priorities [36,37]. By attending to the priorities of the youth, the study materials became more grounded in their real-life experiences, which improved the relevancy and responsivity of our research. Researchers and clinicians seeking to engage the historically underserved population of youth with IDD in behavioral health research can leverage CeNR and methods from our protocol to enhance the accessibility and inclusivity of their studies.

## CRediT authorship contribution statement

**K.L. Berg:** Writing – review & editing, Writing – original draft, Supervision, Resources, Project administration, Methodology, Investigation, Funding acquisition, Conceptualization. **D Herrman:** Writing – review & editing, Writing – original draft, Project administration, Methodology, Conceptualization. **L Bernard:** Writing – review & editing, Writing – original draft, Methodology, Funding acquisition, Conceptualization. **C.S Shiu:** Writing – review & editing, Writing – original draft, Methodology, Funding acquisition, Conceptualization. **I Mihaila:** Writing – review & editing, Supervision, Project administration, Investigation. **C Arnold:** Writing – review & editing, Project administration, Funding acquisition, Conceptualization. **K Acharya:** Writing – review & editing, Methodology, Funding acquisition, Conceptualization. **T.R.G Gladstone:** Writing – review & editing, Methodology, Funding acquisition, Conceptualization. **C Danguilan:** Writing – review & editing, Writing – original draft, Validation, Methodology. **H Gussin:** Writing – review & editing, Methodology, Funding acquisition, Conceptualization. **P Perez:** Writing – review & editing, Project administration, Methodology, Funding acquisition, Conceptualization. **A Herrman:** Writing – review & editing, Methodology, Conceptualization. **S Aaron:** Writing – review & editing, Methodology, Funding acquisition, Conceptualization. **A Thornton:** Writing – review & editing, Supervision, Project administration. **M Gerges:** Writing – review & editing, Methodology, Funding acquisition, Conceptualization. **C Patriarca:** Writing – review & editing, Supervision, Project administration. **J.J Pak:** Writing – review & editing, Methodology, Conceptualization. **B.W Van Voorhees:** Writing – review & editing, Supervision, Resources, Project administration, Methodology, Investigation, Funding acquisition, Conceptualization.

## Declaration of competing interest

The authors declare that they have no known competing financial interests or personal relationships that could have appeared to influence the work reported in this paper.

## Figures and Tables

**Table:** 

Welcome & Introductions	
**Project Updates**	
**Workshops for Caregivers and Teens – 2024 topics**	
**TEAMS Workbook update**	
**Conference Presentations**	
**Input on New Research Questions**	
**Future Meetings:** **February 21, 2024** **May 8, 2024**	

**Figure:**
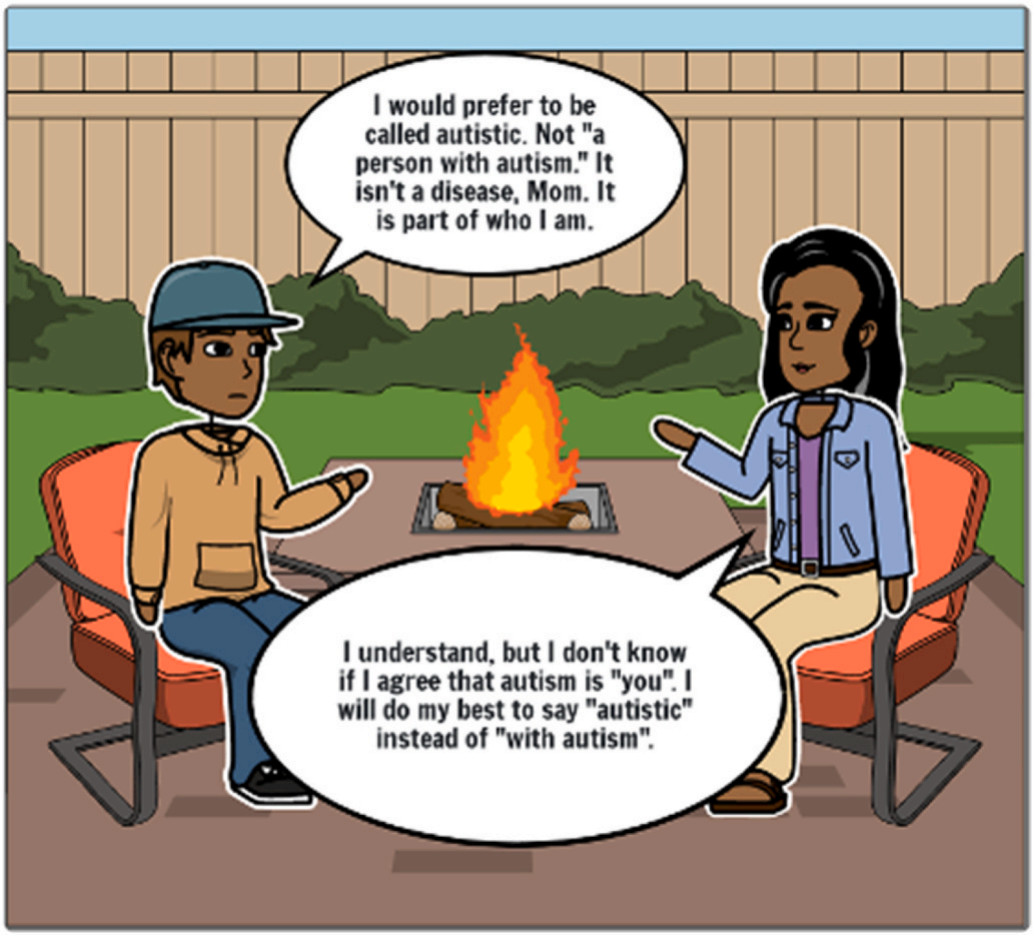


**Figure:**
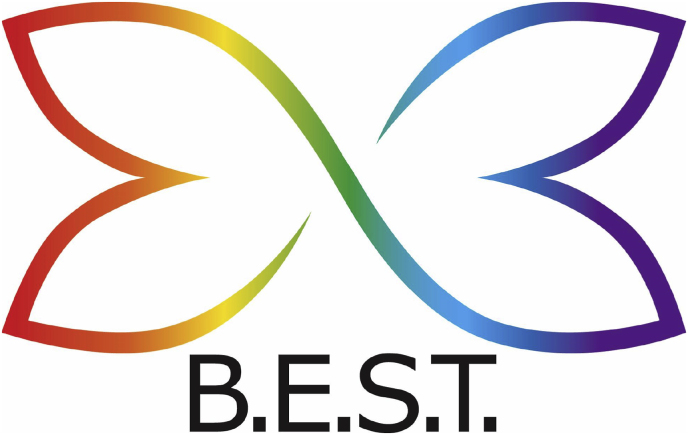


## Data Availability

Data will be made available on request.

## APPENDIX A

1. Youth Selected LOGO
2. Comics Co-Designed and Written by Youth
3. Accessible Agenda

**PCORI BEST Youth Advisory Committee meeting Wednesday, November 8th, 2023 7:00pm**.

**Next meeting: Wednesday, February 21st, 2024 7:00pm**.

## Appendix A

2. Comics Co-Designed and Written by Youth.
